# Repurposing Antihypertensive Drugs for the Prevention of Glaucoma: A Mendelian Randomization Study

**DOI:** 10.1167/tvst.11.10.32

**Published:** 2022-10-20

**Authors:** Jingjing Liu, Shuang Li, Yang Hu, Shizheng Qiu

**Affiliations:** 1 Eye Hospital, the First Affiliated Hospital of Harbin Medical University, Harbin, Heilongjiang, China; 2 Beidahuang Industry Group General Hospital, Harbin, China; 3 School of Computer Science and Technology, Harbin Institute of Technology, Harbin, China

**Keywords:** antihypertensive drugs, glaucoma, systolic blood pressure, diastolic blood pressure, mendelian randomization

## Abstract

**Purpose:**

Several antihypertensive drugs have been used for the treatment of glaucoma. However, the effect of hypertension and antihypertensive drugs on glaucoma is still unclear.

**Methods:**

Leveraging large-scale genome-wide association study summary statistics for glaucoma (Ncase = 4737, Ncontrol = 458,196), blood pressure (BP) (N = 422,771), and intraocular pressure (IOP) (N = 31,269), the genetic correlation and causal relationship of genetically assessed IOP, systolic blood pressure (SBP), diastolic blood pressure (DBP), and 12 types of antihypertensive drugs with glaucoma were evaluated using linkage disequilibrium score (LDSC) regression, univariate mendelian randomization (MR), and multivariable MR.

**Results:**

LDSC results showed a suggestive association of glaucoma with SBP (*R_g_* = 0.12, *P* = 0.0076) and DBP (*R_g_* = 0.17, *P* = 0.02). In univariate MR, genetically elevated BP in participants was not identified to lead to an increased glaucoma risk (SBP: odds ratio [OR], 1.05 [95% confidence interval {CI}, 0.91–1.21]; *P* = 0.52; DBP: OR, 1.07 [95% CI, 0.93–1.23]; *P* = 0.34). The results of univariate MR were replicated in multivariable MR (SBP: OR, 0.95 [95% CI, 0.71–1.29]; *P*  = 0.75; DBP: OR, 1.13 [95% CI, 0.85–1.51]; *P*  = 0.41). Furthermore, there was insufficient evidence to suggest that antihypertensive drugs were associated with glaucoma.

**Conclusions:**

Together, controlling BP may not help prevent and treat glaucoma, and antihypertensive drugs may neither treat nor worsen glaucoma.

**Translational Relevance:**

Treating with antihypertensive drugs should not be used as an intervention for patients with glaucoma.

## Introduction

Glaucoma is the second-leading cause of blindness and the first leading cause of irreversible blindness, affecting more than 80 million people worldwide.^1^^,^^2^ Although current treatments for glaucoma still focus on reducing intraocular pressure (IOP), glaucoma is a multifactorial disease, and the single-treatment approach delays the treatment of other glaucoma patients with non-high IOP.^3^^–^^5^

Several antihypertensive drugs, such as beta-blockers, angiotensin-converting enzyme inhibitors, and calcium channel blockers, have been used to treat glaucoma.^3^^,^^6^^–^^9^ Doctors who take this view of medication believe that high blood pressure (BP) may impair the blood supply to the optic nerve, and therefore the capillary pressure of the ciliary body caused by BP will increase IOP.^10^^–^^12^ However, there have been ongoing debates concerning the therapeutic effect of antihypertensive drugs on glaucoma.^13^^–^^15^ Chong et al.^16^ investigated the correlation between different types of antihypertensive drugs and the thickness of retinal nerve fiber layer and ganglion cell intra-plexus layer in the Asian population and found that antihypertensive drugs might damage the health of retinal ganglion cells. In addition, evidence from the UK Clinical Practice Research Datalink database also supported that glaucoma patients receiving beta-blocker adjuvant therapy were associated with more subsequent visits and hospitalizations.^15^ Nevertheless, Horwitz et al.^3^ investigated 2,689,434 participants aged 40 to 95 years living in Denmark, and the results supported the effect of antihypertensive drugs in preventing the onset and development of glaucoma.

These associations may not be causation, because previous observational studies with relatively small sample sizes may have been confounded by other potential risk factors. Mendelian randomization (MR) is a statistical method that uses genetic variants as instruments to assess potential causal relationship between risk factors and disease outcome.^17^^,^^18^ Importantly, the genotypes of single-nucleotide polymorphism (SNP) instruments are established at birth and will not be altered by confounding factors.^17^ Meanwhile, reverse causality is avoided.

**Figure 1.**
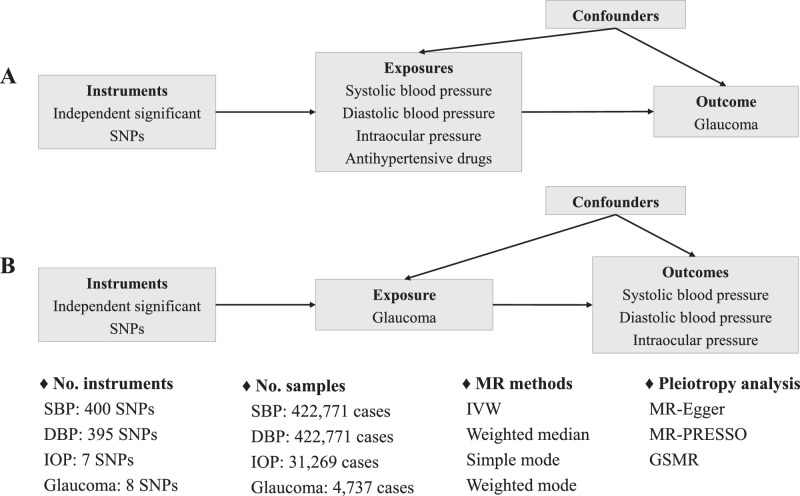
Study design. The study was a MR analysis. In step 1, the genetic correlations of the risk factors with glaucoma were estimated using cross-trait LDSC regression. In step 2, the causal relationship of IOP, SBP, DBP, and 12 antihypertensive drug classes with glaucoma were investigated using univariate MR. In step 3, the causality of IOP, SBP, and DBP with glaucoma were investigated using multivariable MR.

In this study, we investigated the genetic correlation and causal association of genetically assessed IOP, systolic blood pressure (SBP), diastolic blood pressure (DBP), and 12 antihypertensive drug classes with glaucoma using cross-trait linkage disequilibrium score (LDSC) regression and two-sample MR. Assessment of these potential risk factors for glaucoma may have direct public health and clinical implications for the prevention and treatment of glaucoma. A conceptual framework is shown in Figure 1.

## Methods

### Data Sources

Genome-wide association studies (GWAS) summary statistics for glaucoma was obtained from the UK Biobank (UKBB). UKBB is a large national and international health resource of genetic and phenotype data from ∼500,000 volunteers aged 40 to 69 recruited in the United Kingdom between 2006 and 2010.^19^ The glaucoma cases were defined as self-reported glaucoma status in response to the question “Has a doctor told you that you have any of the following problems with your eyes?”^19^^,^^20^ The control group included the non-cases and excluded subjects who reported “Prefer not to answer” or “Do not know.”^19^ GWAS summary statistics for BP was also obtained from UKBB. Participants underwent a standardized portfolio of phenotypic tests (two BP measurements taken seated after two minutes rest).^19^

GWAS summary statistics for IOP were obtained from 12 European ancestry cohorts from the International Glaucoma Genetic Consortium.^20^ Both eyes of participants were measured to calculate the mean IOP.^20^

The SNP instruments used to assess the effect of the protein targets of antihypertensive drugs on glaucoma were obtained from the study by Walker et al.^21^ They collected 12 antihypertensive drug classes, including adrenergic neuron blockers, alpha-adrenoceptor blockers, angiotensin-converting enzyme inhibitors, angiotensin-II receptor antagonists, beta-adrenoceptor blockers, calcium channel blockers, centrally acting antihypertensives, loop diuretics, potassium-sparing diuretics (PSDs) and aldosterone antagonists, renin inhibitors, thiazides and related diuretics, and vasodilator antihypertensives in the British National Formulary, identified the corresponding genes of the protein targets of these drugs, and used a Genotype-Tissue Expression project to map SNP instruments to each protein target (Supplementary Table S1).^21^^–^^23^

We removed rare variants (MAF < 0.05) and only focused on autosomal chromosomes (excluding the HLA region) in this study. All the participants of above studies were of European ancestry, and more details are shown in the original studies or Table 1.^19^^–^^21^^,^^24^ The study approval and the written informed consent had been obtained in the original studies.

**Table 1.** Details of GWAS Summary Statistics

Trait	Source	No. of Participants (Cases) and Ethnicity	No. of SNP Instrument
Glaucoma	UKBB^19^	4737 European individuals	8
IOP	IGGC^20^	31,269 European individuals	7
SBP	UKBB^19^	422,771 European individuals	400
DBP	UKBB^19^	422,771 European individuals	395
Antihypertensive drugs	Walker et al.^21^	NA	5, 10, 1, 5, 10, 23, 8, 3, 3, 2, 11, 12

IGGC, International Glaucoma Genetics Consortium.

### Genetic Correlation of IOP, SBP and DBP With Glaucoma

LDSC regression was used to estimate the genetic correlation of IOP, SBP and DBP with glaucoma.^25^ Precomputed LD scores were derived from 1000 Genome Project reference panels.^25^^,^^26^ SNP heritability and sample overlap were also estimated.^25^ The statistically significant association is defined to be *P* < 0.05/5 = 0.01 after multiple testing.

### Univariate MR Analysis

Causal relationships of genetically assessed IOP, SBP, DBP and 12 antihypertensive drug classes with glaucoma were investigated using MR analysis, including inverse variance weighted (IVW), weighted median, simple mode and weighted mode. A total of 7400 and 395 independent genome-wide significant SNPs (*P* < 5E-08, *r*^2^ < 0.3) were selected from summary statistics for IOP, SBP and DBP as instruments to perform two-sample MR. Furthermore, generalized summary-data-based mendelian randomization (GSMR) analysis was used as a complementary approach to MR, which used the HEIDI-outlier to filter out SNP outliers that might be pleiotropic.^17^^,^^27^ MR-Egger and MR pleiotropy residual sum and outlier (MR-PRESSO) were conducted as sensitivity analyses.^17^^,^^28^^,^^29^ In addition, reverse MR analysis was performed. The statistically significant association was defined as *P*  <  0.05/3 = 0.0167 after multiple testing.

### Multivariable MR Analysis

Multivariable MR was used to assess the causal estimates of several genetically related risk factors on disease outcome.^17^^,^^30^ When all three risk factors (IOP, SBP and DBP) were simultaneously used as exposures, multivariable MR only selected independent SNPs significantly associated with one of the exposures as instruments, and performed the MR model in linear regression.^17^^,^^30^

### Statistical Analysis

All the statistical analyses in this study were based on R 4.0 or Python 2.7 environment and were conducted using packages “two-sample MR,” “MR-PRESSO,” GCTA (genome-wide complex trait analysis), and LDSC. Wald test was used to calculate 2-tailed *P* values. The statistically significant association was defined as *P* < 0.05 after adjusting for multiple testing.

## Results

### Genetic Correlation of IOP and BP With Glaucoma

From cross-trait LDSC regression, we found strong evidence of the genetic correlation of glaucoma with IOP (*R_g_* = 0.65, *P* = 3.16E-09) and SBP (*R_g_* = 0.12, *P* = 0.0076) and a suggestive correlation with DBP (*R_g_* = 0.17, *P* = 0.02) (Table 2). Meanwhile, IOP was suggestively associated with SBP (*R_g_* = 0.09, *P* = 0.018) but not DBP (*R_g_* = 0.04, *P* = 0.21) (Table 2). These results indicated a high proportion of shared genetic associations between glaucoma and IOP or SBP and a potential correlation between IOP and SBP.

**Table 2.** SNP-Based Genetic Correlation Estimated From LDSC Regression

Trait 1	Trait 2	*Rg*	Z	*P*	LDSC Intercept
Glaucoma	IOP	0.652 (0.11)	5.92	**3.16E-09**	0.0096 (0.0066)
Glaucoma	SBP	0.124 (0.046)	2.67	**0.0076**	−0.0164 (0.008)
Glaucoma	DBP	0.108 (0.046)	2.32	0.02	−0.0114 (0.0084)
IOP	DBP	0.0512 (0.041)	1.26	0.21	−0.0051 (0.0079)
IOP	SBP	0.094 (0.04)	2.37	0.018	−0.0013 (0.0073)

The statistically significant association was defined as *P*  <  0.05/5 = 0.01 after adjusting for multiple testing.

### Univariate MR

**Figure 2.**
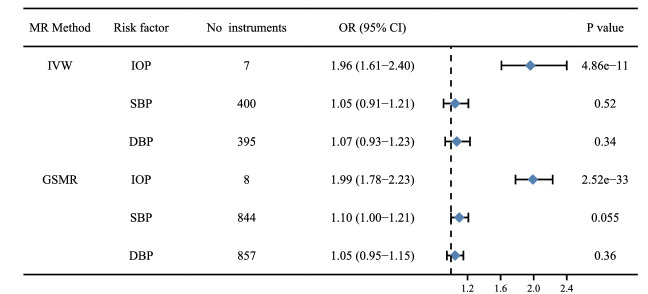
Causal association of genetically assessed risk factors on glaucoma. The statistically significant association was defined as *P*  <  0.05/3 = 0.0167 after adjusting for multiple testing.

**Figure 3.**
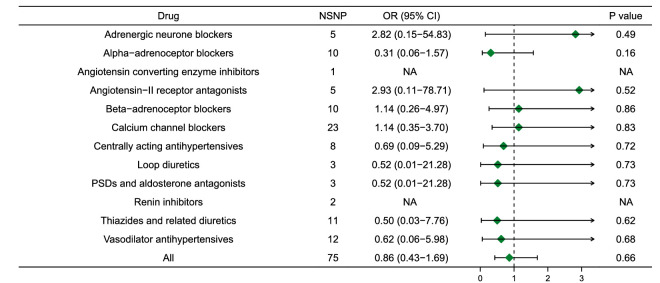
Association of 12 antihypertensive drugs with glaucoma in MR analysis. The statistically significant association was defined as *P*  <  0.05/12 = 0.0042 after adjusting for multiple testing.

Five MR methods, including IVW, weighted median, simple mode, weighted mode and GSMR, provided similar results (Fig. 2, Supplementary Tables S2–S5). For each per-unit (mm Hg) increase in IOP, glaucoma risk increased 1.96-fold (OR, 1.96; 95% confidence interval [CI], 1.61–2.40; *P*  = 4.86E-11) (Fig. 2). Genetically assessed SBP and DBP were not associated with increased risk of glaucoma. MR-Egger and MR-PRESSO testing detected some outliers, with no significant difference in the causal estimates before and after adjusting for the outliers (Supplementary Tables S2–S6). In addition, there was no evidence that 12 types of antihypertensive drugs had a causal association with the risk of glaucoma using drug target MR analysis (Fig. 3).^21^^,^^31^

### Multivariable MR

Given the potential genetic overlap of IOP and BP, multivariable MR analysis was implemented to estimate the independent causal effect of each exposure (IOP, SBP, and DBP) on glaucoma. Interestingly, only IOP was associated with glaucoma (OR, 1.78; 95% CI, 1.10–1.26; *P*  = 3.13E-06), whereas SBP (OR, 0.95; 95% CI, 0.71–1.29; *P*  = 0.75) and DBP (OR, 1.13; 95% CI, 0.85–1.51; *P*  = 0.41) were not (Supplementary Table S7).

### Reverse MR Analysis

**Figure 4.**
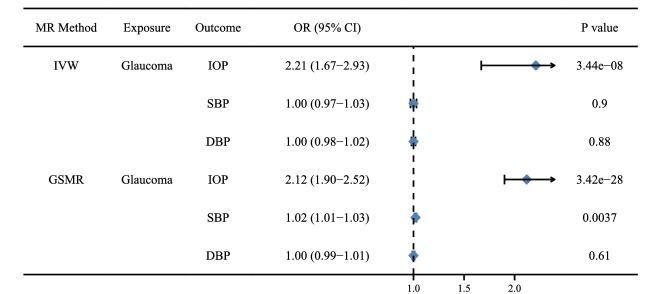
Causal association genetically assessed glaucoma on IOP, SBP and DBP. The statistically significant association was defined as *P*  <  0.05/3 = 0.0167 after adjusting for multiple testing.

To verify whether glaucoma patients were at potential risk for hypertension and ocular hypertension, we implemented a reverse MR analysis. High IOP was significantly associated with glaucoma (OR, 2.21; 95% CI, 1.67–2.93; *P* = 3.44E-08), and glaucoma patients might have a slightly increased risk of developing elevated SBP in only GSMR analysis (OR, 1.02; 95% CI, 1.01–1.03; *P* = 0.0037) (Fig. 4, Supplementary Tables S8–S12).

## Discussion

Previous studies have disputed the role of BP and antihypertensive drugs in glaucoma.^3^^,^^6^^–^^8^^,^^13^^–^^15^ Some studies suggest that increased ocular ciliary blood flow and capillary pressure in hypertensive patients leads to increased aqueous humor secretion or poor aqueous humor circulation.^32^ In addition, increased external scleral venous pressure leads to a decrease in aqueous humor, further increasing IOP.^32^ Zhao et al.^33^ conducted a large meta-analysis of 60 observational studies on glaucoma risk in hypertensive patients and showed that the pooled relative risk for primary open-angle glaucoma in hypertensive patients was 1.16 (95% CI, 1.05–1.28) compared with patients without a history of hypertension. Their results support the role of elevated BP in increasing IOP. However, observational studies may have overlooked some important confounding factors, such as age, socioeconomic status, lifestyle, and genetic factors associated with BP. In addition, other studies suggest that high BP and BP medications may not be associated with or even increase the risk of glaucoma.^34^^,^^35^ Thus more accurate glaucoma prevention measures are needed in patients with hypertension and patients treated with antihypertensive medication.

In this study, we conducted genetic correlation and MR analyses to investigate the associations of IOP, BP, and antihypertensive drugs with glaucoma risk. The results provided support for the association of IOP with glaucoma; however, the risk of glaucoma might not increase with a higher level of SBP or DBP. Importantly, antihypertensive drugs may have a limited effect on glaucoma, neither contributing to treatment nor harming the patient's health.

Based on large-scale genetic data, our study has many strengths. First, MR analysis provides almost unbiased causal estimates using large-scale GWAS summary statistics from a sample of more than 400,000 individuals and avoids the difficulty in judging confounding factors and reverse causality in traditional observational studies.^17^ It is well known that the MR design is based on the following three assumptions: (1) instrumental variables are directly related to exposure (e.g., BP); (2) instrumental variables cannot affect outcome (e.g., glaucoma) through a pathway other than exposure (i.e., there is no pleiotropic effect of instrumental variables); and (3) instrumental variables are unrelated to confounding factors. If assumptions are violated (especially horizontal pleiotropy in genetic variants), causal estimates will be greatly biased. Thus we used four MR methods (IVW, weighted median, simple mode, and weighted mode) to ensure the robustness of the results, and three pleiotropic test methods (MR-Egger, MR-PRESSO, and GSMR) to ensure the MR assumption is not violated. The results showed that there was no pleiotropy in SNP instruments, and several MR analysis methods also gave similar results. Second, all of the participants in this study were of European descent, therefore minimizing bias due to population stratification. Finally, BP and IOP were adjusted for potential genetic overlap using multivariable MR analysis, so accurate estimates of the risk of glaucoma could be obtained.

This study also has several limitations. First, glaucoma cases for this study were from self-reported glaucoma in UK Biobank. It is widely known that UKBB is a cohort study in which individuals are sampled from the general population, so the number of cases of glaucoma was relatively small.^19^^,^^36^ Self-reported disease status may miss some suspected cases of glaucoma and mistakenly include some cases of ocular hypertension.^37^^–^^40^ Second, the number of instrumental variables for glaucoma is slightly small, and the heritability to explain glaucoma is limited. Third, our results only apply to European lineages, and the effect of BP on glaucoma in other lineages remains to be studied. Finally, in this study, the therapeutic target genes of antihypertensive drugs were selected as instrumental variables to calculate the contribution of antihypertensive drugs to the treatment of glaucoma. It is difficult to take into account information about when and how long the antihypertensive drugs were taken, and when the glaucoma was diagnosed. Assessing the role of BP on glaucoma from the genetic perspective may explain only part of the role of BP on glaucoma.

## Conclusions

Genetic evidence shows that lowering BP via the protein targets of antihypertensive drugs is unlikely to affect glaucoma risk. We provide valuable public health and clinical advice that the use of blood pressure–lowering and the use of antihypertensive drugs as interventions in patients with glaucoma remains open to question.

## Supplementary Material

Supplement 1
